# Work–Family Conflict Among Employees and the Self-Employed Across Europe

**DOI:** 10.1007/s11205-015-0899-4

**Published:** 2015-02-21

**Authors:** Anne Annink, Laura den Dulk, Bram Steijn

**Affiliations:** Department of Public Administration, Erasmus University Rotterdam, Rotterdam, The Netherlands

**Keywords:** Employment relationship, Europe, Self-employed, State support, Work–family conflict

## Abstract

This article examines the level of work–family conflict of self-employed persons, a changing but neglected group in work–life research, compared to employees in Europe. Differences between the two groups are explained by looking at job demands and resources. The inclusion of work–family state support makes it possible to examine differences between countries. Multilevel analysis has been applied to data from the European Social Survey (ESS 2010). The results show that job demands and resources operate differently for employees and the self-employed. The relationship between employment type and WFC is mediated mainly by job demands such as working hours, working at short notice, job insecurity and supervisory work. The results also reveal variation across countries that cannot be explained by state support, signalling the need for a more complete understanding of WFC from a cross-national perspective.

## Introduction

Combining work and family responsibilities is an issue for many workers today, whether employed or self-employed. Workers perform different roles in the work and family domains. When these roles are mutually incompatible in some way, a form of inter-role conflict arises (Greenhaus and Beutell 1985: 77). This may take the form of work-to-family conflict or family-to-work conflict. This article focuses on work-to-family conflict (WFC), since research shows that work tends to conflict more with family life than vice versa (Frone 2003).

Workers—especially women and/or parents—often believe that self-employment will ease the pressure of combining work and family (Eurofound 2007). Self-employment enables workers to combine income, flexibility and control over their work and childcare (Sullivan and Meek 2012). Research shows that the self-employed may have job resources that improve their ability to balance work and family life, such as autonomy, flexibility, skill utilization and job security derived from the feeling that their future is in their own hands (Parasuraman and Simmers 2001). Job autonomy in particular is related to lower stress and WFC (Prottas and Thompson 2006). Despite the benefits of self-employment, the associated job demands—long working hours, work intensity, demanding and stressful work, insecurity and precariousness—may also create tensions and lead to WFC (Parasuraman and Simmers 2001). In addition, stress arising from the present economic crisis may negatively impact employees´ ability to reconcile family life and paid employment (Gregory et al. 2013).

These findings show that available job resources may not offset the job demands self-employed persons face in combining work and family. Research findings on the self-employed’s WFC are inconclusive. Some studies show that the self-employed experience more WFC than employees (Frone 2000; Nordenmark et al. 2012; Parasuraman and Simmers 2001), while others argue the other way around (Craig et al. 2012; Prottas and Thompson 2006).

To shed light on previous contradictory results regarding the WFC of the self-employed, this article compares their WFC to that of employees from a cross-national perspective. WFC is an important issue because it is an indicator for health, well-being, quality of life and duration of self-employment (OECD 2011; Williams 2004). The importance of preventing WFC is acknowledged by the European Union, who sets guidelines for support. However, although governments are giving increasingly attention to reconciling paid employment and parenting, research shows that arrangements for the self-employed lag behind those for employees and that they differ across European countries (Pedersini and Coletto 2010; Annink et al. 2015). Recently, researchers on WFC of employees have started to include work–family state support (i.e. leave and childcare) in their research models, but the results on the effectiveness of these policies are inconclusive across countries. Countries with relatively generous state support such as Sweden also score high on experienced work–family conflict, for instance (Strandh and Nordenmark 2006; Van der Lippe et al. 2003).

The aim of this article is to explicate the multilevel mechanism underlying the relationship between employment type and WFC. It uses the Job Demands-Resources (JD-R) model to identify how WFC arises in different occupational settings. The JD-R model assumes that although every occupation may have its own job-stress risk factors, these factors can be grouped into two general categories (i.e. job demands and job resources). The JD-R model is thus overarching and can be applied to various occupational settings, such as employment or self-employment (Bakker and Demerouti 2007). This study extends earlier research on WFC by answering the following research questions: (a) do job demands and resources mediate the relationship between employment type and WFC? and (b) do job demands and resources operate differently for employees and the self-employed?

The present study answers these questions by analysing data from the European Social Survey 2010 on employees and the self-employed in 17 European countries using a multilevel design. The European Union has set guidelines for work family state support for employees and for the self-employed. However, within the boundaries of these guidelines, countries formulate their own policies. By analyzing the influence of state support on individual work-to-family-conflict, we acknowledge that workers in different European countries may have different experiences. First, a combined model is estimated in order to examine whether job demands and resources mediate the relationship between employment type and WFC. Next, two separate analyses for employees and the self-employed enable comparison of the coefficients of job demands and job resources, indicating whether each one works out differently for the two employment groups.

This study advances the existing literature on WFC in two important ways. First, earlier research on WFC has been conducted among employees working in large organizations within single countries. This article adds to current (contradictory) research findings by examining how and why the WFC of employees and the self-employed differ. It investigates and compares the underlying mechanisms of the JD-R model for both employees and the self-employed (see also Nordenmark et al. 2012; Prottas and Thompson 2006; Parasuraman and Simmers 2001). Second, until now, state support has not been taken into account in research on WFC of the self-employed and we do not know whether this is beneficial for them or not. Although the European Union intends to protect the self-employed and their spouses by introducing maternity leave, this may be counterproductive, since it is very difficult to combine maternity leave with running a business (Neergaard and Thrane 2011).

The research model and hypotheses in this study result from the theoretical model discussed in the next section. This is followed by the methods applied, after which the multi-level results are presented. The article ends with the conclusion and a discussion.

## Theoretical Model and Hypotheses

The aim of this article is to identify mechanism underlying the relationship between employment type and WFC. We are interested in the specific functioning of work related resources and demands which are available to both employees and the self-employed, but might work out differently for both employment types. In a recent study on the wellbeing and work–life balance of the self-employed, Nordenmark et al. (2012) used the Job Demand-Control model (Karasek 1979). They found that high levels of job control and job demands create conflict between work and family and are negatively related to work–life balance. However, they also showed that the level of job control hardly varies among the self-employed. This is not unexpected, as job control is related to individual responsibility and effort, which can be seen as inherent to self-employment (Beugelsdijk and Noorderhaven 2005).

In this article we therefore use the Job Demands-Resources (JD-R) model, which builds on the Job Demands-Control model but allows us to include also other resources that impact WFC (Demerouti et al. 2001). The JD-R model is often used in work–family research on employees to shed light on the specific occupational conditions that either cause problems (i.e. job demands that conflict with family life) or help solve them (i.e. resources that support a good work–family balance) (Bakker and Demerouti 2007; Bianchi and Milkie 2010; Voydanoff 2005).

*Job resources,* such as support, are enriching and lead to work engagement and commitment; they enable workers to meet goals that reduce WFC directly and indirectly (Xanthopoulou et al. 2007). According to the JD-R model, job resources are important in their own right for achieving work-related goals and by stimulating personal growth and development, but they are also important in dealing with job demands (Bakker and Demerouti 2007). A job resource can become a buffer and reduce the stressor or the perceptions and cognitions evoked by such stressors (Bakker and Demerouti 2007).

*Job demands,* such as working hours, working at short notice, job insecurity and being a supervisor, are “those physical, social, or organizational aspects of the job that require sustained physical and/or psychological effort” (Bakker and Demerouti 2007: 312). High job demands may exhaust employees’ resources and cause the work domain to have a negative impact on the family domain (i.e. WFC).

So far, the JD-R approach has focused mainly on individual job characteristics and less on the way the broader environmental and national context impacts people’s resources and demands. Only recently researchers have started to include work family state support, such as leave and childcare, as a resource into their research models (see: Stier et al. 2012; Ruppanner 2013). However, these studies are limited to support for employees. In 2010, the right to maternity leave for the self-employed was introduced, which should allow self-employed women to interrupt their occupational activity for at least 14 weeks during pregnancy or motherhood. Within the boundaries of EU guidelines, countries are free to formulate their own policies. Including state support for employees and the self-employed, allows us to examine the effect on individual WFC and to explain differences across countries.

The model below shows that job demands and job resources at the individual level and state support at the country level are expected to mediate the relationship between employment type (being employee or self-employed) and WFC. This is formulated in hypothesis 1a, 1b and 1c. Second, the model seeks to identify the mechanism that underlies the relation between employment type and WFC, via the inclusion of job demands and resources. As explained in the introduction, the self-employed have different job demands and resources and work–family state support than employees, which may explain differences in WFC. This is formulated in hypothesis 2, 3 and 4.

Research on employees shows that many resources may reduce WFC, but especially social support appears to be an important job resource. A meta-analysis of Kossek et al. (2011) shows that both the type and the source of social support an employee receives matters for WFC. Social support can be general or work–family specific. In this article we focus on the latter. Ayman and Antani (2008) argue that it is important that people who are active in multiple life domains (such as work and family) also have large and diverse support networks which can provide them with support. Demerouti et al. (2004) show that low social support in the home situation increases WFC. According to Adams et al. (1996), family members and spouses have a unique opportunity to provide both emotional and instrumental support to the worker outside of the work environment. Selvarajan et al. (2013) show that emotional support provided by the spouse has the beneficial effect of promoting overall emotional well-being which may have helped in dealing with conflict originating in both family and work domains. Based on these findings and the JD-R model, we expect the degree of spousal and social support is related to type of employment. The degree of support might in turn influences the level of WFC. The first hypothesis tests whether spousal and social supports have a mediating effect on WFC. We will explore differences between both types of employment and the underlying mechanisms further from hypothesis two onwards.

### **H1a**

Job resources (spousal and social support) mediate the effect of employment type on WFC

Several job demands contribute to WFC in the context of employment. Especially long work hours, a heavy workload and work pressure have been found to be important predictors for WFC (Greenhaus and Beutell 1985; Grzywacz and Marks 2000; Frone 2003). Furthermore, there is strong evidence that workers who regard their current employment as insecure are more likely to experience physical problems and psychological distress and less vigor at work, less job satisfaction, and less work–family enrichment (Burgard et al. 2009; Cheng et al. 2013). The relationship between being a supervisor and WFC is less clear. Prottas and Thompson (2006) show that small business ownership is a double-edged sword: the greater pressure associated with ownership of a small business detracts from the advantages of having autonomy. Those working as independent contractors appear to reap the benefits of greater autonomy. Being a supervisor might be experienced as a resource due to more autonomy, but is also associated with more WFC (Voydanoff 2005). Hypothesis 1b tests whether job demands play an important role in governing the relationship between employment type and WFC.

### **H1b**

Job demands (working hours, working at short notice, job insecurity and supervision) mediate the effect of employment type on WFC

As previous research shows, not only individual job demands and resources influence individual’s experiences, but also national policies and institutional arrangements might reduce employed women’s and men’s sense of conflict (Stier et al. 2012; Ruppanner 2013). Research on employees indicates leave and childcare as important resources. Childcare for children under the age of three is explicitly recognized as helping families reconciling care and employment (Gornick and Meyers 2003; Steiber 2009). Ruppanner (2013) notes that indices for work scheduling, school scheduling, and early childhood education and care showed no clear effects on work–family conflict for working parents. The same author therefore suggests that research should explicitly focus on leave, since it plays an important role in explaining parent’s conflict between work and family. Leave is meant to support caregiving while allowing parents to remain in employment. However, cross-country variation in enrolment rates for childcare reflects variation in the public provision of childcare, in parental leave systems, in other incentives for women to work, and in culture and family structures (OECD 2009). We therefore include both leave and childcare arrangements. Although the EU has introduced maternity leave for the self-employed in 2010, arrangements lag behind those for employees and (Pedersini and Coletto 2010; Annink et al. 2015) and might cause unintended effects (Neergaard and Thrane 2011). The next hypothesis tests whether state support explicates the process that underlies the relationship between employment type and WFC.

### **H1c**

State support (leave and childcare) mediates the effect of employment type on WFC

Next, assuming that job demands and resources and state support do have a mediating effect, certain job demands, job resources and state support might have a different (stronger or weaker) effect on WFC for the self-employed, due to their specific work characteristics. It is important to compare those effects, because the specific work characteristics of the two employment groups might make certain job demands, job resources and state support more or less important and make their effect on WFC stronger or weaker.

An example of such a work characteristic is job autonomy, which has been reported as important job resources (Bakker and Demerouti 2007). However, it was impossible to include job autonomy in this study, since almost all self-employed persons decide how their daily work is organized, make their own policy decisions and choose the pace of work. Our analysis showed that there is no variance on job autonomy among the self-employed, probably because it is a defining job characteristic of this subsample.

**Fig. 1:**
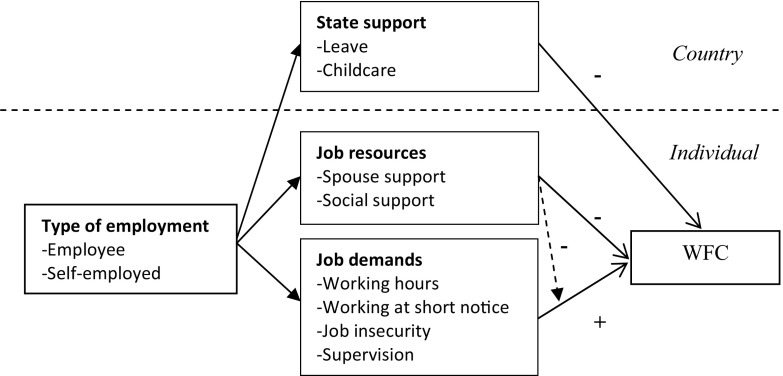
The connections between employment relationship, job demands and resources and state support and WFC

Other job resources might work out differently for both employment groups. Regarding social support, research shows that the self-employed report lower levels of social support than employed workers because they lack co-worker support (Taris et al. 2008; Tuttle and Garr 2009), although they can compensate by joining professional networks (Koster and De Vries 2011). The self-employed also lack supervisor support, which is negatively related to WFC (Matthews et al. 2010). Because of this relatively lonely work situation, the impact on WFC of social support outside work is expected to be stronger for the self-employed. Gunnarsson and Josephson (2011) demonstrated an association between an active social life and good health for entrepreneurs, which might reduce stress. In Fig. 1, the dotted arrow therefore visualizes the expected buffer effect of social support for the self-employed.

### **H2a**

Social support outside work has a stronger negative effect on WFC for the self-employed than for employees

Spousal support is also indicated as an important resource for the self-employed (Md-Sidin and Sambasivan 2008). Eddleston and Powell (2012) show in their study that male entrepreneurs appear to be experience less conflict between work and family when their spouse takes care of the family and household. Due to their lack of co-worker and supervisor support, spousal support is expected to have a stronger negative effect for the self-employed.

### **H2b**

Spousal support has a stronger negative effect on WFC for the self-employed than for employees

Among the job demands, long working hours are often mentioned as causing conflict. Working long hours might be incompatible with other life domains and may lead to WFC (Parasuraman and Simmers 2001; Tuttle and Garr 2009). However, the self-employed report more “passion for work” and higher work engagement (Gorgievski et al. 2010). As a result, the self-employed might experience working long hours as less demanding than employees because they see working as an investment in their business or as an extended hobby.

### **H3a**

Working long hours has a stronger positive effect on WFC for employees than for the self-employed

Another job demand is working overtime at short notice. Since the self-employed determine their own working hours, they are more flexible than employees about planning and rearranging their work schedule at short notice. Therefore, they are likely to experience less WFC as a result of having to work at short notice.

### **H3b**

Working at short notice has a stronger positive effect on WFC for employees than for the self-employed

Cheng et al. (2013) found that the self-employed experience WFC due to job insecurity. Job control did not seem to buffer this effect. Job insecurity is likely to have a greater impact on the WFC of the self-employed, since they are solely responsible for their income and more vulnerable to precariousness.

### **H3c**

Job insecurity has a stronger positive effect on WFC for the self-employed than for employees

Supervising other employees may also be more demanding for the self-employed because they are fully responsible for them. Supervisors in an organization might have smaller teams or departments to manage and share their responsibilities with co-workers, which may be experienced as less demanding.

### **H3d**

Supervision has a stronger positive effect on WFC for the self-employed than for employees

Regarding state support, countries differ in the extent to which they offer childcare and leave to employees and the self-employed. Stier et al. (2012) found that the widespread availability of day care centres for young children allowed employed parents to better balance their work and family demands. Organizations may offer corporate childcare as an employee benefit (financial support, referral services or workplace crèche) or as part of their CSR policy. Because the self-employed have no employer support, the effect of state childcare support on WFC is expected to be stronger for the self-employed. Nordenmark et al. (2012) suggests that childcare may mitigate the negative job control-related effects of self-employment on reconciling work and family.

### **H4a**

Childcare support has a stronger negative effect on WFC for the self-employed than for employees

The effect of leave on WFC is more complicated. Ruppanner (2013) demonstrates that mothers report less work–family conflict in countries with more expansive family leave policies. Pedersini and Coletto (2010) show that leave arrangements are generally less extensive for the self-employed and vary across European countries. In contrast to Ruppanner (2013), Neergaard and Thane (2011) argue that the effects of maternity leave may be different for employees and the self-employed because the latter cannot take maternity leave and receive a state income allowance while keeping their business going. This means that being a new mother is irreconcilable with owning a business. Therefore, our last hypothesis is:

### **H4b**

Leave has a stronger negative effect on WFC for employees than for the self-employed

## Methodology

### Data and Design

This study uses two data sources. First, extensive desk research resulted in a scored overview of state support, i.e. leave and childcare, for employees and the self-employed across Europe (see Appendix 1). Second, data from the European Social Survey (ESS) was used to investigate differences in WFC between employed and self-employed persons. Round 5 of the ESS was conducted in 2010 and included a module on work, family and well-being. It also made it possible to examine the impact of state support and variance in WFC across 17 selected European countries: Belgium, Bulgaria, Czech, Denmark, Estonia, Finland, France, Germany, Hungary, The Netherlands, Norway, Poland, Portugal, Slovenia, Spain, Sweden and the United Kingdom.

### Sample

In this study, the labour force includes all persons aged 15–65 who normally worked at least 12 h per week, overtime included, and selected either “employee” or “self-employed” as their main activity. In the rotating module Work, Family and Well-being, the questions only concerned respondents living with a spouse or partner. In testing the impact of leave arrangements and childcare on WFC, the present study looked only at employees and the self-employed with children living at home, since these arrangements are only relevant for and used by parents. People working in a family business are not considered self-employed in this study. Unlike other businesses, a family business is built to pass on to the children; planning and strategic decisions are thus negotiated with family members (Astrachan and Shanker 2003).

All variables were box-plotted, after which outliers were removed. This left a total sample of 6,192 respondents, divided into employee (N = 5,399) or self-employed (N = 793). The distribution of our sample across countries is shown in Appendix 1.

The employees (87 %) in this sample worked 40 h a week (overtime included) on average in sectors such as education, public administration and defence, education and human health services. 32 % were supervisors; 52 % were male and 48 % female. Their average age was 42. The self-employed (13 %) in this sample worked 49 h a week on average. The largest share worked in retail, personal services and crop and animal production. 44 % were supervisors; 66 % were male and 34 % female. Their average age was 44.

### Measurement

#### Dependent Variable

*W* *FC* was composed of four questions (Cronbach’s alpha 0.73) on a scale of 1 (never) to 5 (always). The questions were: how often do you “…find that your partner or family gets fed up with the pressure of your job?”, “…keep worrying about work problems when you are not working?”, “… feel too tired after work to enjoy the things you would like to do at home?” and “… find that your job prevents you from spending time with your partner or family?”

#### Job Demands

Based on the theoretical framework, working hours, working at short notice, job insecurity and supervising employees were included as job demands. *Working hours* is defined as the number of hours a respondent normally works a week (in his or her main job), including overtime. *Working at short notice* is measured by how often the respondent has to work overtime at short notice on a scale of 1–7. *Job insecurity* measures how often the respondent had to do less interesting work, accept a pay cut, work shorter hours and was less secure in his or her job in the past 3 years, on a scale of 1–5 (Cronbach’s alpha 0.64). Respondents could work in a s*upervisory position* (1) or not (0). The question posed to respondents was “In your main job, do you have any responsibility for supervising the work of other employees?”

#### Job Resources

*Spousal support* and *social support* were indicated as important job resources. *Spousal support* indicates the number of hours the respondent’s partner spends doing household chores. S*ocial support* is measured by how often respondents participate in social activities compared to other people of their age, on a scale of 1–7.

#### State Support

*Leave* and *childcare* were included as resources for handling family demands in the JD-R model. Leave refers to maternity and paternity leave, which are birth-related leaves available to mothers or fathers and often accompanied by wage-related benefits, and parental leave, which refers to longer leave periods that enable parents to care for young children at home, either immediately after birth or in the subsequent period (Misra et al. 2011). Leave (0–9) is the sum of maternity, paternity and parental leave, based on duration and payment as recorded in the country notes of local experts (Moss 2010). For each type of leave, 0 was assigned to countries offering no leave, 1 to entitlement but unpaid, 2 to entitlement either at a low flat rate or at <66 % of earnings, and 3 to entitlement paid to all parents at more than 66 % of earnings for some or all of the leave. The score concerns the minimum statutory entitlements, irrespective of payment ceilings. For comparison purposes, the study did not take into account whether leave is transferable or whether it is a family or individual entitlement. The scores for the self-employed are based on Moss (2010), the European Commission (2010) online database on Social Protection and Social Inclusion, and local government websites. The leave variable represents the situation in April 2010. The ESS round 5 fieldwork was gathered between October 2010 and May 2011, which made it possible to analyse representative relationships between leave arrangements and respondents’ answers. Analysis of the effect of *childcare* on WFC was based on the *enrolment rates*, which indicate the country’s percentage of children aged 0–2 in formal care, such as childcare centres and registered child-minders. Childcare is accessible for both employees and the self-employed, although it might be more expensive for the latter. As explained before, employees are more likely to receive (a higher) financial benefit or compensation.

Data issues make cross-country comparison difficult. Enrolment rates may be underestimated in countries where a significant proportion of childcare is private (e.g. Ireland). Overestimation may occur in countries where young children may be enrolled in several part-time programmes and counted twice (OECD 2009). Enrolment rates fluctuate over time due to amendments in compulsory employers’ contributions, for example. Enrolment rates may also be influenced by leave; they may be lower in countries with extensive leave arrangements. Nevertheless, the enrolment rate is the best measure for this study. Since this article compares countries, it is interested in the childcare measure with the most variation. Lastly, research on childcare in Eastern Europe is limited (Szelewa and Polakowski 2008), but the availability of enrolment rates for children aged 0–2 made it possible to include them.

### Control Variables

The individual-level control variables considered are *gender, age, sector* and *feelings about household income* (scale 1–4). Based on gendered work and family roles, resources and demands are assumed to work out differently for women than for men. Earlier studies found that the male and female self-employed experience WFC differently (Eddleston and Powell 2012; Ruppanner 2013). Nordenmark et al. (2012) have shown that the self-employed experience a poorer work–life balance than the employed, but this outcome is more prominent among men. However, when control and demands at work are held constant for the self-employed and the employed, self-employed women experience a significantly better work–life balance than employed women, and self-employed men experience a similar work–life balance to employed men. Since these mechanisms have been explored elsewhere, gender differences lie beyond the scope of this study. The study controlled for household income, since respondents who have difficulty managing their household budget are likely to experience more WFC. At the country level, the study controlled for unemployment rate as a percentage of the labour force, since the economic situation and job opportunities could influence the employment type.

### Descriptive Statistics

In Table 1, the asterisks (*) in the self-employed’s mean column resulting from *t* tests indicate whether the self-employed’s scores differ significantly on these variables from the employees’. The descriptive statistics show that the self-employed are more likely than employees to be male and older. On the one hand, they earn a higher household income, but on the other their unemployment rate is also higher. They work longer hours, more often at short notice, do more supervisory work and experience more job insecurity, but also receive more spousal and social support. The state allows them less leave than employees. Finally, the self-employed experience more work–family conflict than employees.

**Table 1:** Mean scores and differences between employees and self-employed on dependent and independent variables (job demands, job resources and state support)

Variable	Min	Max	Employees			Self-employed			All		
			N	M	SD	N	M	SD	N	M	SD
Dependent											
WFC	1	5	5,341	2.30	0.75	788	2.56***	0.75	6,129	2.33	0.75
Job demands											
Working hours	12	130	5,399	40.48	9.42	793	48.8***	14.88	6,129	41.54	10.64
Working at short notice	1	7	5,355	2.68	1.54	778	3.72***	1.82	6,133	2.82	1.61
Job insecurity	1	5	5,399	1.35	1.49	768	1.82***	1.60	6,086	1.41	1.51
Supervision	0 (no)	1 (yes)	5,399	0.32	0.47	793	0.44***	0.50	6,192	0.34	0.47
Job resources											
Spousal support	0	100	5,110	14.40	13.10	758	17.56***	14.46	5,868	14.80	13.31
Social support	1	7	5,399	4.79	1.47	793	4.94**	1.45	6,192	4.81	1.46
State support											
Leave	0	9	5,399	6.47	1.61	793	4.76***	2.77	6,192	6.10	1.96
Enrolment rates	2.2	65.7	5,399	32.58	18.66	793	32.84	18.46	6,192	32.61	18.63
Control variables											
Gender	0 (m)	1 (f)	5,399	0.48	0.50	793	0.34***	0.47	6,192	0.46	0.50
Age	19	65	5,399	42.33	8.50	793	44.14***	8.18	6,192	42.57	8.44
Household income	1	4	5,383	3.12	0.78	792	3.24***	0.68	6,175	3.14	0.77
Unemployment rate	3.2	18	5,399	8.27	3.42	793	8.90***	3.83	6,192	8.35	3.46

* *p* < 0.05; ** *p* < 0.01; *** *p* < 0.001

The country structure is not taken into account in this table

### Methods

The study tested the hypotheses by means of both descriptive and explanatory analyses. Stata 12 was used to estimate multilevel analyses. First, a combined two-level model was estimated in which individuals (1) were clustered hierarchically within countries (2). This made it possible to examine whether job demands, job resources and state support mediate the relationship between employment type and WFC. Second, two multilevel models for employees and the self-employed were estimated. To see whether the effect of job demands, job resources and state support on WFC varies by employment group, the B coefficients and the confidence interval were compared per variable. Regression coefficients (B), standard errors (S.E.), levels 1 and 2 variance explained by the models, and the −2 Log likelihood as an indicator of model fit are reported for each of the models (Table 2).

**Table 2:** Results of multilevel linear regression of WFC on employees’ and the self-employed’s job resources and demands and state support

	Model 1		Model 2		Model 3		Model 4		Model 5		Model 6		Model 7	
	B	SE	B	SE	B	SE	B	SE	B	SE	B	SE	B	SE
Constant	2.331***	0.033	2.710***	0.074	2.860***	0.085	1.707***	0.089	1.726***	0.110	1.727	0.018	1.80	0.171
Individual characteristics														
Employment relationship (1 = self-employed)			0.254***	0.028	0.247***	0.029	−0.008	0.028	−0.006	0.031	−0.006	0.031	−0.007	0.031
Gender (female = 1)			−0.057**	0.020	−0.031	0.023	0.152***	0.022	0.151***	0.022	0.151***	0.022	0.151***	0.022
Age			−0.002	0.001	−0.002	0.001	−0.001	0.001	−0.008	0.001	−0.001	0.001	−0.001	0.001
Sector			0.000	0.000	0.001	0.000	0.009*	0.000	0.001*	0.000	0.001*	0.000	0.001*	0.000
Household income			−0.110***	0.014	−0.010***	0.015	−0.120***	0.014	−0.116***	0.014	−0.116***	0.014	−0.115***	0.014
Job resources														
Spousal support					0.002**	0.001	0.001	0.001	0.001	0.001	0.001	0.001	0.001	0.001
Social support					−0.042***	0.007	−0.036***	0.007	−0.035***	0.007	−0.035***	0.007	−0.051*	0.025
Job demands														
Working hours							0.014***	0.001	0.14***	0.001	0.014***	0.001	0.012***	0.003
Working at short notice							0.128***	0.006	0.128***	0.006	0.129***	0.006	0.112***	0.021
Job insecurity							0.053***	0.006	0.053***	0.006	0.053***	0.006	0.089***	0.020
Supervision (1 = yes)							0.123***	0.020	0.124***	0.020	0.124***	0.020	0.095	0.070
State support														
Leave									0.001	0.008	0.001	0.001	0.001	0.009
Enrolment rates									−0.001	0.001	−0.001	0.001	−0.001	0.001
Country characteristic														
Unemployment rate											−0.000	0.007	−0.000	0.007
Buffering effect of job resources														
Social support *working hours												0.000	0.001	
Social support *working at short notice												0.003	0.004	
Social support *job insecurity												−0.008	0.004	
Social support *supervision												0.006	0.014	
Individual variance	0.74	0.01	0.73	0.01	0.73	0.01	0.66	0.01	0.66	0.01	0.66	0.006	0.67	0.006
Country variance	0.13	0.025	0.13	0.02	0.11	0.02	0.09	0.02	0.09	0.02	0.09	0.02	0.09	0.02
−2 Log likelihood		13,770.6		13,460.6		12,790.7		11,444.7		11,444.2		11,444.2		11,438.9

*Source* European Social Survey (2010). N = 6,492; * *p* < 0.05; ** *p* < 0.01; *** *p* < 0.001

## Results

### The Mediating Effect of the JD-R Model

To examine whether the job demands, job resources and state support mediate the relationship between employment type and WFC, Table 3 presents a two-level model that combines the employee and self-employed samples. In models 1–7, individual characteristics, job resources, job demands, state support, country characteristics and interaction effects were added one by one to examine when differences between employees and the self-employed would become non-significant. The first individual characteristic is the dummy variable employment relationship (1 = self-employment). This is the main variable of interest, because it shows whether the employment relation significantly influences WFC after controlling for job demands and resources.

**Table 3:** Results of multilevel linear regressions of work–family conflict on employees’ job demands, job resources and state support

	Employees									
	Model 1		Model 2		Model 3		Model 4		Model 5	
	B	SE	B	SE	B	SE	B	SE	B	SE
Constant	2.30***	0.03	2.64***	0.078	2.78***	0.090	1.60***	0.097	1.65***	0.143
Gender (female = 1)			−0.040	0.020	−0.010	0.024	0.176***	0.024	0.175***	0.024
Age			−0.002	0.001	−0.002	0.001	−0.001	0.001	−0.000	0.001
Sector			0.006	0.000	0.001	0.000	0.001*	0.000	0.001*	0.000
Household income			−0.091***	0.015	−0.082***	0.015	−0.110***	0.015	−0.107***	0.015
Job resources										
Spousal support					0.002**	0.001	0.001	0.001	0.001	0.001
Social support					−0.039***	0.008	−0.032***	0.007	−0.031***	0.007
Job demands										
Working hours							0.015***	0.001	0.015***	0.001
Working at short notice							0.132***	0.007	0.132***	0.007
Job insecurity							0.049***	0.007	0.049***	0.007
Supervision (1 = yes)							0.144***	0.021	0.144***	0.022
State support										
Leave									−0.005	0.016
Enrolment rates									−0.001	0.001
Individual variance	0.73	0.01	0.73	0.007	0.73	0.000	0.67	0.007	0.67	0.007
Country variance	0.13	0.03	0.13	0.03	0.13	0.020	0.10	0.020	0.09	0.02
−2 Log likelihood		11,918.7		11,715.8		11,135.2		10,049.9		10,024.4

* *p* < 0.05; ** *p* < 0.01; *** *p* < 0.001

In models 2 and 3, this employment relationship dummy differs significantly between employees and the self-employed in terms of WFC. However, after controlling for job demands in model 4, this difference is no longer significant (B = −0.008). Models 5–7 also include state support, country characteristics and interaction effects, none of which are significant. Model 4 is the best fitting model. This implies that mainly job demands explain differences in WFC between employees and the self-employed.

Regarding the hypotheses, 1a is unsupported; differences between employees and the self-employed remain after controlling for job resources. Spousal support and social support do not mediate the relationship between employment relation and WFC. Regarding the interaction effects, findings indicate that the included job resources have no buffering effects on job demands. The best fitted model 4 shows that WFC differences between employees and the self-employed can be explained by job demands. Hypothesis 1b is therefore supported: job demands (working hours, working at short notice, job insecurity and supervision) mediate the effect of employment type on WFC. At the country level, state support and childcare cannot explain WFC differences between employees and the self-employed.

### Effects of Job Demands and Resources

To answer the second research question “Do job demands and resources operate differently for employees and the self-employed?”, the study estimated two separate models for employees and the self-employed. In Table 3, models 1–5 represent the employee models and in Table 4, models A to E represent the self-employed models. In these models, the main interest lies in the B’s of the independent variables (i.e. the differences between them).

**Table 4:** Results of multilevel linear regressions of work–family conflict on the self-employed’s job demands, job resources and state support

	Self employed									
	Model A		Model B		Model C		Model D		Model E	
	B	SE	B	SE	B	SE	B	SE	B	SE
Constant	2.55***	0.035	3.54***	0.205	3.80***	0.230	2.47***	0.232	2.48***	0.234
Gender (female = 1)			−0.217***	0.057	−0.223***	0.063	−0.020	0.059	−0.021	0.059
Age			−0.002	0.003	−0.004	0.003	−0.004	0.003	−0.004	0.003
Sector			0.000	0.001	0.000	0.001	0.001	0.001	0.001	0.001
Household income			−0.256***	0.040	−0.231***	0.040	−0.202***	0.038	−0.190***	0.039
Job resources										
Spousal support					0.002	0.002	0.000	0.001	0.000	0.002
Social support					−0.058**	0.019	−0.059***	0.017	−0.060**	0.017
Job demands										
Working hours							0.020***	0.002	0.013***	0.002
Working at short notice							0.107***	0.015	0.109***	0.015
Job insecurity							0.066***	0.015	0.067***	0.015
Supervision (1 = yes)							−0.014	0.051	−0.015	0.050
State support										
Leave									−0.004	0.009
Enrolment rates									−0.002	0.001
Individual variance	0.75	0.019	0.72	0.018	0.71	0.019	0.63	0.017	0.63	0.017
Country variance	0.10	0.040	0.10	0.04	0.08	0.047	0.05	0.052	0.02	0.113
−2 Log likelihood		1,782.1		1,724.1		1,629.6		1,381.9		1,380.4

* *p* < 0.05; ** *p* < 0.01; *** *p* < 0.001

### Job Resources

Model 1 and A are the empty models. Model 2 and B show that the control variables gender and household income are negatively related to WFC for both employees and the self-employed. Self-employed persons who have negative feelings about their household income are especially prone to WFC. Female workers experience more WFC than male workers. In model 3 and C, WFC was regressed for the job resources social support and spousal support. Social support appears to have a negative effect on WFC for both employees and the self-employed. This means that the more often individuals participate in social activities, the less WFC they experience. The B’s and the confidence intervals (not shown) of social support do not differ substantially between employees and the self-employed. Hypothesis 2a is therefore unsupported. Contrary to hypothesis 2b, spousal support has a positive effect on employee WFC. This means that the more hours the employee’s partner works in the household, the more WFC he or she experiences. Contrary to the findings of Eddleston and Powell (2012), spousal support has no significant effect on WFC for either the male or female self-employed in this study.

### Job Demands

Regarding job demands, the results in model 4 and D show that all job demands have a significant positive effect on WFC for both employees and the self-employed, except for supervision for the self-employed. Comparing the effects between the two employment groups, working at short notice and being a supervisor both have a stronger effect on WFC for employees than for the self-employed. Hypothesis 3a is supported regarding working at short notice. To illustrate the strength of the relationship between working hours and WFC: an employee who “never” experiences WFC would have to work 14 h more in order to experience WFC “sometimes”. For a self-employed person this is 17 h.

Hypothesis 3b is confirmed regarding job insecurity; both employees and the self-employed experience more WFC if they had to do less interesting work, take a pay cut, work shorter hours and had less security in their job in the past 3 years. This effect is stronger for the self-employed. On the other hand, being a supervisor has a significantly positive impact on WFC only for employees. This means that employees with supervisory tasks experience more WFC than non-supervisory employees. Being a supervisor has no significant effect on WFC for the self-employed.

### State Support

In contrast to the expectations, but in line with Table 2, state support and childcare show no significant effects on the WFC of employees and the self-employed. Hypothesis 4a and 4b are therefore unsupported.

## Discussion

In this study, the European Social Survey is used to compare the WFC of self-employed persons and employees across 17 European countries. This final section summarizes and discusses the results of the study.

First, we found that the self-employed experience more WFC than employees. The JD-R model developed by Demerouti and Bakker (2007), which has been shown to be overarching and applicable to various occupational settings, was used to examine whether this difference could be explained by job demands and resources. Second, it appeared that the relationship between employment type (i.e. employee or self-employed) and WFC is mainly mediated by job demands such as working hours, working at short notice, job insecurity and being a supervisor. Our results support Nordenmark et al. (2012), who found that high levels of job demands are negatively related to work–life balance. Job resources (i.e. spousal and social support) did not mediate the relationship between employment type and WFC, either directly or indirectly. A major contribution of this article to current literature is the unravelling of the underlying mechanism of WFC by showing how the effects of job demands and resources differ for employees and the self-employed and by showing that we should focus on job demands primarily to understand these differences. Third, this article shows that social support appeared to be an important resource for the self-employed, perhaps because they lack supervisor and co-worker support (Matthews et al. 2010; Taris et al. 2008; Tuttle and Garr 2009). This finding suggests that it might be worthwhile for the self-employed to take part in social activities more often. Contrary to expectations, spousal support had a positive effect on the WFC of employees. It may be that the spouse working at home instead of in a paid job puts pressure on the spouse who earns the main household income. It could also be that employees find it important to do household chores themselves, and that they feel less competent and powerful as a result of their spouse’s assistance (Martire et al. 2002). Job demands showed the largest effects on WFC for both employment groups, especially working at short notice. However, it might be a little easier for the self-employed to reschedule their tasks at short notice, perhaps preventing conflict. Job insecurity had a large effect on the WFC of the self-employed. The employees in this sample experienced being a supervisor as the heaviest demand, while it had no effect on the WFC of the self-employed. This implies that the results of this study might be relevant for small business owners as well.

### The JD-R Model

Although the job demands and resources model proofed to be successful in helping to explain WLB and to distinguish between negative and positive work-related factors, little attention is paid to the impact of the wider societal and institutional context (Drobnič and León 2014). This article included widely available state support as a resource, because research on employees shows that this might reduce employed women’s and men’s sense of conflict (Abendroth and Den Dulk 2011; Stier et al. 2012; Ruppanner 2013). Although according to the European Parliament, the economic and physical vulnerability of pregnant self-employed workers and pregnant spouses made it necessary to introduce maternity leave for the self-employed in 2010, some authors doubt the intended effects for the self-employed (Neergaard and Thrane 2011). In this study, state support was not found to have any mediating effect. Differences in WFC cannot be explained by leave, childcare and the unemployment rate. However, the results do demonstrate that some of the variance in WFC between employees and the self-employed can be explained at the national level. Therefore, a more complete understanding of the causes of WFC can be achieved when individual and country characteristics are studied in cross-level combinations. Explanations might be found in the cultural norms, gender ideology and a supportive environment.

Cultural norms and expectations affect the way existing policies are used and thereby the division of domestic work, care for children and paid work (Budig et al. 2012). The actual utilization of policies may show more about work–family outcomes than formulated policies. The effect of state support might also be non-significant due to differences in gender ideology. An unequal division of paid and unpaid work might be perceived as conflicting in some countries, while it is not in a more traditional gender culture. Gender expectations might also explain the positive effect of spousal support on WFC in this study. Research shows that people who agree that “men should take as much responsibility as women for the home and children” are less likely to feel conflict (Steiber 2009). Future research could test whether the self-employed maintain more egalitarian attitudes than employees. Lastly, research shows that a supportive work environment stimulates the uptake and effectiveness of state support (Den Dulk et al. 2010). Research on employees shows that general organizational support and support from the supervisor moderates the relationship between national paid leave and work–family conflict (Allen et al. 2014). These findings stress the importance of further studying the role of (spousal) support for the self-employed in work-to-family conflict. The strength of family networks and solidarity are also expected to play an important role (Sarceno 2011).

### Limitations and Future Research

Future research could first look into the non-significance of state support by reconsidering the definition of state support used in this article. In this study, it refers to leave and childcare available to parents with children under age three, while the sample is composed of parents with children up to age 18. Job autonomy could not be included as a resource is this study due to its limited measurement in the European Social Survey. Scholars have different theories about the relationship between job autonomy and WFC for employees and the self-employed. On the one hand, job autonomy offers more opportunities to cope with stressful situations, which is crucial for health and wellbeing (Karasek 1998). On the other hand, individuals with autonomous work often experience more time pressure (Voydanoff 2005). In short, the high level of job autonomy in self-employment does not necessarily lead to less WFC (Drobnič and Guillén Rodríguez 2011). Future research might also reconsider the definition of autonomy as it is used currently. The concept of autonomy is often associated with independence or individualism, while when acting autonomously, people are fully behind their own actions—they feel choiceful and integrated in behaving. Ryan and Deci (2011: 49) define being autonomous as “acting from one’s intrinsic interests and from internalized values and regulations”. Autonomy could be more meaningful in comparative research if operationalized in terms of volition, willingness, and endorsement. Second, the European Social Survey excluded the self-employed from questions about work pressure, social support at work and flexibility, for example. Third, prior research argues that the actual characteristics of the work performed might have a more important effect on WFC than the employment status as such (Hytti et al. 2013). Although we acknowledge the wide variance in self-employment contexts, no distinction could be made between categories of self-employed due to their small number per country. Another important distinction for future research would be between opportunity-driven and necessity-driven self-employment. Binder and Coad (2013) found that only opportunity-driven individuals who move voluntarily from regular employment into self-employment experience an increase in life satisfaction. It might be that the opportunity-driven self-employed experience less WFC than the necessity-driven self-employed. The finding that the self-employed experience more WFC than employees could be related to the timing of this survey, which was conducted in 2010 at the height of the economic crisis. The crisis has been stressful for many self-employed persons, which could increase their WFC. It would be interesting to examine the effect of the economic crisis on the WFC of the self-employed. Now that this study has made the differences between employees and the self-employed somewhat clearer, research into the work–family issues of the self-employed can become a world in itself.

## Appendix 1

See Table 5.

**Table 5:** Number of respondents and national-level characteristics per country

	Country	Number of employees	Number of self-employed	Total no. of respondents	Maternity Emp.	Paternity Emp.	Parental Emp.	Leave employees	Maternity leave Se.	Paternity leave Se.	Parental leave Se.	Leave self-employed	Enrolment rates (0–2) in formal care (2008)	Unemployment rate (2009)
1	Belgium	327	49	376	3	3	2	8	2	0	0	2	48.4	7.9
2	Bulgaria	326	28	354	3	3	3	9	0	0	0	0	14.6	6.8
3	Czech	360	53	413	1	0	2	3	3	0	0	3	2.2	6.7
4	Denmark	334	52	386	2	2	2	6	2	2	0	4	65.7	6.0
5	Estonia	303	36	339	3	1	2	6	3	0	3	6	17.5	13.8
6	Finland	282	58	340	3	3	2	8	3	3	3	9	28.6	8.2
7	France	287	35	322	3	3	2	8	3	3	0	6	42.0	9.5
8	Germany	451	60	511	3	0	2	5	0	0	3	3	17.8	7.8
9	Hungary	307	24	331	3	3	2	8	2	0	3	5	8.8	10.0
10	Netherlands	291	40	331	2	3	2	7	2	0	0	2	55.9	3.7
11	Norway	356	29	385	3	1	2	6	3	0	3	6	51.3	3.2
12	Poland	328	78	406	3	0	2	5	3	3	0	6	7.9	8.1
13	Portugal	247	35	282	3	3	2	8	3	3	3	9	47.4	10.6
14	Slovenia	285	22	307	3	2	2	7	3	2	3	8	33.8	5.9
15	Spain	316	88	404	2	2	1	5	2	2	0	4	37.5	18.0
16	Sweden	282	55	337	3	3	2	8	3	3	3	9	46.7	8.3
17	United Kingdom	317	51	368	2	2	1	5	0	0	0	0	40.8	7.6
	Total	5,648	844	6,492										

Key leave arrangements: 0 = no statutory entitlement. 1 = statutory entitlement but unpaid. 2 = statutory entitlement, paid but *either* at low flat rate *or* at <66 % of earnings *or* not universal *or* for less than the full period of leave. 3 = statutory entitlement, paid for some or all of leave to all parents at more than 66 % of earnings

*Sources* Moss (2010), OECD (2013), UNECE (2013), European Commission (2010) and national government websites
